# Brief interventions for cannabis use in emerging adults: protocol for a systematic review, meta-analysis, and evidence map

**DOI:** 10.1186/s13643-018-0772-z

**Published:** 2018-07-25

**Authors:** Jillian Halladay, Tashia Petker, Allan Fein, Catharine Munn, James MacKillop

**Affiliations:** 1 Department of Health Research, Evidence, and Impact, McMaster University, Hamilton, Ontario Canada; 2 Michael G. De Groote Centre for Medicinal Cannabis Research, McMaster University/St. Joseph’s Healthcare Hamilton, Hamilton, Ontario Canada; 3 Peter Boris Centre for Addictions Research, McMaster University/St. Joseph’s Healthcare Hamilton, 100 West 5th Street, Hamilton, Ontario L8S 4L8 Canada; 4 Department of Psychiatry and Behavioural Neurosciences, McMaster University, Hamilton, Ontario Canada

**Keywords:** Cannabis, Marijuana, Brief intervention, Motivational interviewing, Motivational enhancement, Emerging adult, Student, Meta-analysis, Systematic review

## Abstract

**Background:**

Rates of cannabis use are highest during emerging adulthood (age 18–25), with the prevalence of near daily and daily increasing among this age group. Emerging adults are clinically challenging in terms of harmful cannabis use due to perceptions of high rates of peer use, social acceptance, and low risk of harm. Brief interventions to increase awareness and promote motivation to change are therefore particularly important for this age group. There is existing evidence on the effectiveness of brief interventions for alcohol in emerging adults, but it is not clear if comparable evidence is present for cannabis. The objective of this systematic review is to summarize and critically appraise the existing literature of brief interventions for cannabis use both narratively, to describe the content and delivery of existing interventions, and meta-analytically, to determine the aggregated efficacy of these interventions on cannabis use and other outcomes (e.g., other substance use, mental health, help-seeking behaviors, and academic and occupational outcomes).

**Methods:**

A systematic search of randomized controlled trials, quasi-experimental trials, and pre-post designs will be conducted in the following electronic databases: MEDLINE, EMBASE, Cochrane Central Register of Controlled Trials, Allied and Complementary Medicine, Cumulative Index to Nursing and Allied Health Literature, and PsycINFO. Ongoing trials will be identified using the World Health Organization International Clinical Trials Registry Platform, ClinicalTrials.gov, and Current Controlled Trials. Unpublished trials will be identified using Proquest Dissertations, OpenGrey, Google Scholar, and brief interventions on the Substance Abuse and Mental Health Services Administration webpage. Two authors will independently screen and extract data from articles using a predetermined screening and extraction forms (which will include risk of bias assessments). Calibration exercises will be performed prior to full screening and extraction. Disagreements will be resolved through discussion or consultation with a third reviewer. All studies will be reported narratively, and if appropriate, we will perform random effects meta-analyses with subgroup analyses and meta-regression.

**Discussion:**

Results of this review are expected to provide guidance on the content, delivery methods, and effectiveness of brief interventions for cannabis use to assist post-secondary institutions in identifying brief intervention strategies to implement prior to or in response to legalization.

**Systematic review registration:**

CRD42018085412

**Electronic supplementary material:**

The online version of this article (10.1186/s13643-018-0772-z) contains supplementary material, which is available to authorized users.

## Background

Cannabis is one of the most commonly used psychoactive substances in the world, with use most prevalent among emerging adults (15–24 year olds) [1–5]. Cannabis use has been associated with negative physical and mental health effects in a dose-response fashion, where near daily or daily use is associated with worse outcomes [6]. Harmful levels of use have been increasing among emerging adults in Canada [7, 8] and the United States [1]. Risks of cannabis-related harm are more pervasive for individuals who begin using cannabis during adolescence, with earlier age of first use potentially resulting in long lasting consequences [6]. Given the high prevalence of use and potential for significant impairment, efficacious interventions for high-risk individuals in this age group are a pressing clinical priority.

Emerging adults are neurodevelopmentally vulnerable to the effects of cannabis, as cannabis acts on areas of the brain integral to brain development. The brain is not completely matured until approximately 25 years of age [9]. Chronic, regular cannabis use during adolescence has shown to negatively affect memory, attention, and psychomotor skills [10–12], potentially causing irreversible cognitive impairment [13] resulting in an increased likelihood of fatal car accidents [14], poor academic performance [15–17], and dropping out of school [17] . Additionally, weekly cannabis use or a cannabis use disorder is almost 10 times as likely in people with other mental illness as compared to those without other mental illness [18]; frequent use has been associated with psychotic disorders [14, 19], bipolar disorder [14], personality disorders [20], depression [20–22], anxiety [23], and suicidal ideation and attempts [24]. Overall, cannabis use during emerging adulthood is particularly concerning due to its potential to disrupt neurological, social, emotional, and cognitive development.

Despite the associations between cannabis use and negative health consequences, the perceived risk of using cannabis, both occasionally and regularly, is decreasing among adolescents and emerging adults [25–28] and, subsequently, there has been an increase in social acceptance of cannabis use [29]. This is further compounded by the perception of high-frequency peer use; in North America, emerging adults perceive that about 86% of their peers are using cannabis at least monthly when only about 20% of students actually report using monthly [7, 30]. Additionally, for general mental health concerns, less than half of emerging adults in need receive professional help [31] with common reported barriers to treatment including stigma, embarrassment, problems recognizing symptoms, self-reliance, and importantly, not enough time [32, 33]. These barriers to seeking general mental health services may be magnified for substance use concerns due to the perception of normalcy of problematic behaviors. Therefore, the perception of low-risk, high peer use, and social acceptance make emerging adults a clinically challenging population, which is further compounded by low motivation for change.

Existing literature on interventions targeting post-secondary substance users acknowledges the challenges to engaging this population and, therefore, has focused on brief interventions (BI) or brief motivational interventions (BMIs). There are many different existing definitions for brief interventions but all contain typical ingredients. BIs typically are short in duration [34, 35] and include provision of personalized feedback [36]. Most BIs adopt a motivational interviewing (MI) approach with the primary goal of the BI being to motivate participants to change their behavior, teach behavioral change skills, and connect to services [35, 37–39]. Often, BIs follow the FRAMES model in which the participant receives personalized *feedback*, reinforcement of personal *responsibility*, objective *advice* given non-judgmentally, a *menu of options*, an *empathetic* and accepting listener, and encouragement of *self-efficacy* and confidence [38–40].

Emerging adulthood is a distinct developmental period characterized by instability and exploration, often referred to as extended adolescence, in which individuals typically experience reduced parental monitoring but are not yet fully engaged in the responsibilities and expectations of adulthood [41]. Emerging adults straddle adolescence and young adulthood, sharing some similarities with both developmental periods. A scoping search was performed to identify the general breadth of existing brief intervention literature to inform the specifics of this review. Existing literature on BIs for cannabis use is already limited, and is particularly scarce when focused on emerging adults. Thus, BIs for adolescents and young adults should also be explored to provide a comprehensive picture and clinical relevance of the existing BI literature.

We identified three reviews on BIs for adolescent substance use. Jensen (2011) looked specifically at MI approaches (including BIs and longer duration interventions, 1 to 9 sessions) for adolescent (12–20 years of age) substance use, finding evidence that suggests MI approaches are helpful to reduce general substance use in the short and longer term (significance retained at follow-up) [42]. Barnett (2012) conducted a systematic review on MI interventions for adolescent substance use (mean age < 18.5). Although they did not perform a meta-analysis, they indicate that 67% of the studies result in reductions in some form of substance use outcomes, including marijuana use [43]. A Cochrane review by Carney (2016) found six randomized controlled trials (RCTs) of face-to-face school-based BIs for adolescents (under 19 years of age) who experience negative behavioral consequences from subclinical levels of substance use [44]. Moderate-quality evidence suggests that effects of BIs on cannabis frequency and dependence are not significantly different compared to information provision (health promotion materials and harm reduction information). However, when comparing BIs to assessment only (evaluated on substance use but received no intervention), BIs appear to reduce cannabis frequency (SMD − 0.54 [− 0.77, − 0.31]), although this is based on low-quality evidence and only includes results from two RCTs (*n* = 338).

For emerging adults, we identified one relevant literature review by Dennhardt and colleagues (2013) [45]. This review found one observational study and five RCTs on BIs and examined their effect on substance use outcomes, including cannabis use. They did not perform a systematic search and did not combine results meta-analytically, but concluded that brief motivational interventions demonstrated the most promising results for college students, apart from parent-based interventions, and requires further investigation.

For adults, there is a Cochrane review by Gates et al. (2016) on psychosocial interventions for cannabis use [46]. This study found 23 RCTs of psychosocial interventions (which include CBT, MI, mindfulness, counseling or education contingency management, relapse prevention) for adults with cannabis abuse or dependence or near daily users of cannabis who were seeking treatment for their cannabis use or other adults seeking treatment for cannabis use. Results suggest moderate-quality evidence that individuals who receive a psychosocial intervention use cannabis on fewer days compared to inactive control; low-quality evidence that those receiving an intervention were more likely to report point-prevalence abstinence, fewer symptoms of dependence, and fewer cannabis-related problems compared to inactive control; and very low-quality evidence that individuals receiving interventions use fewer joints per day compared with inactive control. Additionally, interventions longer than four sessions for more than a month appear to demonstrate better outcomes and Cognitive Behavioral Therapy (CBT) appears to produce the largest effects followed by motivational approaches.

There are several methodological and conceptual limitations of existing reviews. Methodologically, one study did not including a systematic search [45], two did not include unpublished literature [42, 43], and two only included RCTs [44, 46]. In regards to the interventions, most did not look at BIs (1–2 sessions) neither as a whole or within a subgroup analysis [42, 43, 46], most were not cannabis specific [42–45], and there were common restrictions based on the delivery location of interventions (i.e., only in secondary school [44], only in person [44, 46], no inpatient [42], only outpatient community [46]). In the two most comprehensive Cochrane reviews, there were participant restrictions based on baseline substance use; Gates required participants to have a minimum amount of cannabis use at baseline and excluded individuals who regularly used or had a substance dependence on a substance other than marijuana or nicotine [46] while Carney excluded any individuals who had any substance dependence [44]. Also, only one review covered a youth population (12–25) and is fraught with other methodologically and conceptual limitations including being outdated [42]. Additionally, the lack of gold standard BI approach for cannabis use means that there is significant variability in the content and delivery methods of existing BIs. This variability increases heterogeneity and limits our ability to determine the salient components of a successful BI.

This review seeks to address these gaps by (1) focusing primarily on cannabis related outcomes, (2) conducting an up-to-date systematic search of all BIs for cannabis, inclusive of all youth populations and study designs, (3) providing comprehensive descriptions of the contents and delivery methods of existing BIs, and (4) performing multiple subgroup analyses based on content and delivery methods to try to determine the most important and effective components of a BI.

## Objectives

The research question for this review is as follows: In youth aged 15–24, what is the content and effect of existing brief interventions (1–2 sessions) for cannabis use on (a) cannabis-related outcomes, (b) other substance use, (c) help-seeking behaviors, (d) mental health and well-being, and (e) academic and occupational outcomes? Specifically, our aims are as follows:

1. Qualitatively summarize the content and delivery methods of existing BIs cannabis use
2. Summarize and evaluate the quality of existing evidence of cannabis BI for outcomes including (a) cannabis related outcomes, (b) other substance use, (c) mental health and well-being, and (d) academic and occupational outcomes
3. Quantitatively synthesize primary studies
4. Determine key components and delivery methods of successful brief interventions to assist in identifying the ingredients for a gold standard cannabis BI
5. Present an evidence map of BIs for cannabis use across developmental age groups to synthesize existing evidence in a user-friendly format and identify developmental age-specific gaps in the literature

## Methods

### Eligibility criteria

#### Study designs

We will include all RCT designs including parallel-group, crossover, cluster-randomized and factorial design trials, and observational studies including pre-post designs and quasi-randomized trials.

#### Population

The target population is emerging adults (i.e., 15–24 years of age) [5]. This will include individuals both in and outside of school. We will code studies based on context (e.g., secondary school, university, college, treatment, correctional, etc.). Acknowledging the paucity of literature, we will expand our search to any samples that overlap with our pre-identified emerging adult age group (e.g., adolescents age 13–19 or adults 18+). Studies with samples with a maximum age less than 15 and the minimum age greater than 24 will be excluded. This review will not place restrictions on samples based on nature of participation (i.e., voluntary, mandated), presence or absence of other mental health concerns, student status, study origin, ethnicity, or sexuality.

#### Intervention

Brief interventions will be operationalized as 1–2 sessions focused on cannabis use. BIs including FRAMES interventions are commonly defined as a single session. Including a second session constitutes a reasonable expansion of this definition and allows for scenarios in which an individual provides information at one session and receives feedback at a second session. Interventions comprising 3+ sessions will be excluded for falling outside the scope of being BIs. We will include BIs exclusively focused on cannabis use. BIs focused on cannabis use in combination with alcohol use, tobacco use, or general substance use will be excluded. We will include interventions that conduct an additional preliminary or follow-up session for the purpose of data collection and/or behavioral analysis. We will not place restrictions on method of delivery (i.e., in person, online, over the phone).

#### Comparison

Comparison groups can include within-participant pre-post data, wait list control, treatment as usual, or active controls. This will allow for the inclusion of single-arm, two-arm, or multi-armed randomized or observational trials.

#### Outcome

No gold standard instrument currently exists for the screening and assessment of cannabis use. Although there are 25 screening and assessment instruments available with adequate psychometric properties, evidence for the effectiveness of these tools is limited [47]. With this in mind, any instrument capturing aspects of cannabis use (e.g., frequency, problems, dependence, motivation to change) will be included in this systematic review for cannabis use outcomes. We will also collect data on secondary outcomes including all other substance use outcomes, help-seeking behaviors (e.g., accessing services), mental health (both self-report and diagnostic outcomes), and any academic or occupational related outcomes. Data will be collected for all follow-up time points available.

### Search strategy

We will search OVID MEDLINE In-Process (1946 to present), EMBASE (1974 to present), the Cochrane Central Register of Controlled Trials (CENTRAL), Allied and Complementary Medicine (AMED 1985 to present), CINAHL, and PsychInfo (1806 to present). Ongoing trials will be identified using the World Health Organization (WHO) International Clinical Trials Registry Platform, Clinical Trials.gov, and Current Controlled Trials (http://www.controlled-trials.com/). Unpublished trials will be identified using Proquest Dissertations, OpenGrey (up to February 2017), Google Scholar (1st 50 hits for each search), and brief interventions on the Substance Abuse and Mental Health Services Administration (http://nrepp.samhsa.gov/). No restrictions will be placed on language or publication status. We will check abstracts and reference lists of included articles and existing systematic reviews and contact authors for further information and data when appropriate (two attempts will be made). See Additional file 1 for full search strategy in each database and initial hits for each search (will re-run search to ensure up to date).

### Data collection

#### Selection of studies

Articles will be uploaded to Covidence, an online systematic review management software. Two review authors will use Covidence to screen titles and abstracts of identified studies for possible inclusion based on screening forms (see Additional file 2). We will perform a calibration exercise to maximize consistency ensuring good inter-rater reliability operationalized as a Cohen’s kappa of 0.8. After title and abstract screening, all studies that are selected for full-text review (without regard for agreement between screeners) will be screened. Again, two reviewers will independently assess full-texts for inclusion and disagreements will be resolved through discussion or consultation with a third reviewer. Following completion of full-text screening, final inter-rater reliability will be calculated.

#### Data extraction and management

One review author will independently extract data to a Microsoft Excel spreadsheet including study ID, study design, demographic data, risk of bias assessment, intervention description, comparison description, outcomes and measurement tools, summary of author’s findings, and other comments (see Additional file 3 for full extraction content). A second review author will verify the extraction. A calibration exercise will be performed to maximize consistency. Discrepancies will be resolved through discussion or consultation with a third reviewer.

#### Risk of bias of included studies

Risk of bias (RoB) of included RCTs will be assessed using the Cochrane RoB tool at the study level [48]. Each domain will be judged as “low” or “high” RoB and reviewers will avoid judging bias as “unclear.” Observational studies will be assessed with the RoB in non-randomized studies of interventions (ROBINS-I) tool [49]. We will also include the presence of intervention fidelity checks in our RoB assessments under “other” in Cochrane RoB for RCTs and under “bias due to deviations from intended interventions” in ROBINS-I tool for observational studies. Two review authors will independently conduct RoB assessments. Discrepancies in judgments will be discussed and, if a consensus is not met, a third reviewer will resolve the remaining discrepancies.

### Data synthesis, interpretation, and presentation

We will be collecting continuous and dichotomous outcomes, regardless of the measurement tool. We will use mean differences when continuous outcomes are measured similarly (e.g., frequency of cannabis use, motivation to change, academic grades). Standardized mean differences (SMDs) will be used to combine continuous outcomes of similar constructs measured with different instruments (e.g., measures of substance use or mental health symptoms severity). SMDs will be calculated using the inverse variance method in a random effects model to create a uniform measurement scale and allow pooling of effects across studies. Review Manager 5 (RevMan) software will be used to calculate SMDs and 95% confidence intervals (CI), which uses the Hedges’ adjusted g formula [48]. SMDs (Hedges’ g) of 0.2, 0.5, and 0.8 are interpreted as small, medium, and large effects respectively [48, 50]. We will adjust for factorial designs if a particular group (i.e., control) needs to be entered into the same meta-analysis twice by dividing the sample size of the duplicate group in half, keeping means and standard deviations unchanged [48]. For cluster-randomized studies, the data will be adjusted for the clustering effect by using either the reported adjusted post-score means or the intra-class correlation coefficient (ICC) to calculate the associated design effect (DE) and effective sample size [48].

For dichotomous outcomes (e.g., presence or absence of substance or other mental disorder, access of follow-up services, employment or student status), relative risks (RR) and odds ratios (OR) will be combined using the inverse variance method in a random effects model and reported as either a pooled RR or OR (depending on what is more commonly available in the existing literature) with subgroup analyses comparing ORs and RRs to ensure it is appropriate to pool measures [48]. If pooling results is not possible due to insufficient studies (i.e., less than 2), results will be presented narratively.

#### Dealing with missing data

When there is missing data, trialists will be contacted to obtain missing information (two attempts will be made). If we do not hear from trialists, we will calculate or impute missing values. Missing standard deviations (SDs) will be algebraically calculated using standard error, 95% confidence intervals, or *p* values. If missing SDs cannot be calculated algebraically, and baseline and final SDs are known, SDs will be imputed using a correlation coefficient or a conservative estimate of 0.5. We will also perform a sensitivity analysis for studies that only provide complete case data compared to studies that follow intention-to-treat principles. For dichotomous data, if events are missing, we will perform sensitivity analyses considering worst case scenario (i.e., assuming all participants experienced the “bad” outcome) and best case scenario (i.e., assuming all participants experienced the “good” outcome).

#### Assessment of heterogeneity

Heterogeneity between trials pooled for meta-analyses will be assessed by (1) visual inspection of the forest plot, (2) X^2^ test for statistically significance (*P* < 0.1), and (3) I^2^statistic to examine the proportion of between trial differences not due to chance [48]. The I^2^ statistic will be used to determine the proportion of differences that was not due to chance, where values were assessed as ‘might not be important’ (0%–40%), “moderate” (30%–60%), “substantial” (50%–90%), or “high” (75% to 100%) [48]. Due to the overlap in classification of I^2^ categories, a moderate I^2^ in our study will be operationalized as 40% due to the fact that some degree of heterogeneity would be expected between behavioral interventions. Where subgroup analyses have a minimum of 10 studies in the smaller subgroup, we will carry out meta-regression to test subgroup analyses, and multiple meta-regressions (sample size permitting). All pre-specified subgroup analyses will be carried out regardless of heterogeneity.

#### Subgroup analyses and sensitivity analysis

Gates (2016) conducted a fairly comprehensive set of subgroup analyses for psychosocial interventions targeting cannabis use. We will include all subgroup analyses Gates performed with some additional tests: (1) length of the intervention; (2) method of delivery (i.e., in-person compared to online or over the phone interventions); (3) population characteristics including (a) proportion of males, (b) clinical characteristics (dependence, concurrent other psychiatric illness, concurrent non-cannabis substance use, healthy), (c) treatment-seeking compared to non-treatment-seeking participants (including voluntary compared to mandatory participation), (d) patterns of cannabis use history (e.g., duration or levels of use, number of days of use, number of uses per day, age of initiation, route of delivery); (4) content characteristics including (a) personalized feedback regarding frequency, (b) personalized feedback regarding other outcomes, (c) normative feedback, (d) pros and cons, (e) values exercises, (f) mental health screening and/or discussion, (g) decision balance and/or goal setting, (h) provision of additional resource, and (i) adjunctive therapy or booster sessions; (5) study quality (low and high RoB); (6) study design (RCT and observational studies); and for dichotomous outcomes, (7) RR compared to OR, and (8) measurement instrument used. Significant subgroup differences will be identified using a *p* value of < 0.1. All subgroup topics will also be reported descriptively. If enough studies are present, univariate and multivariate meta-regression analyses will be performed. If possible, all analyses will be performed on the total sample of available studies as well as stratified by developmental age (e.g., adolescents, emerging adults, adults).

#### Quality assessment and presentation

The Grading or Recommendations Assessment, Development, and Evaluation (GRADE) approach will be used to assess the quality of the evidence for each outcome to help infer confidence in the review findings and guide future research [51]. Pooled SMD estimates or pooled RR/ORs, along with the GRADE assessment, will be presented in a Summary of Findings table. This protocol follows procedures outlined in the Preferred Reporting Items for Systematic Reviews and Meta-Analysis Protocols (PRISMA-P) statement (see Additional file 4).

We will additionally create an evidence map using a cross-tabular format of BIs for cannabis use across age groups (e.g., adolescents, emerging adults, and adults) to organize the evidence. Miake-Lye et al. (2016) identified cross-tabular formats as the most common way to present evidence “maps” [52]. This evidence map will detail the number and quality of RCTs and observational studies, the combined sample size, the outcomes measured, and the content of interventions stratified by developmental age group.

## Discussion

Results of this systematic review will help to guide the development and refinement of brief interventions targeting cannabis use in the emerging adult population. Identifying the components of effective interventions is critical to inform brief interventions, which can engage youth and promote further help-seeking and behavior change. Given the potential for long-lasting negative effects of cannabis use during emerging adulthood, and current and impending changes in decriminalization and legalization of recreational cannabis in North America, this is an important step in reducing cannabis use and harms. Brief interventions may hold significant potential in terms of providing cost-effective, time-effective, and youth-friendly interventions. This review is essential to help inform effective BI practices that warrant implementation into practice will guide informed future investigations.

## Additional files

Additional file 1: Search strategy. Completed search strategy corresponding with this protocol. (PDF 61 kb)

Additional file 2: Screening form. First draft of the screening form (not yet piloted). (PDF 56 kb)

Additional file 3: Data extraction content. Data that will be extracted from each included full text in this review. (DOCX 92 kb)

Additional file 4: PRISMA-P checklist. Completed PRISMA-P checklist applied to this protocol. (PDF 165 kb)

## Abbreviations

BI: Brief intervention
CBT: Cognitive behavioral therapy
CI: Confidence interval
GRADE: Grading or recommendations assessment, development, and evaluation
MET: Motivational enhancement therapy
MI: Motivational interviewing
RoB: Risk of bias
ROBINS-I: Risk of bias in non-randomized studies of interventions
SMD: Standardized mean difference
