# Supplementary material for: Brief interventions for cannabis use in emerging adults: protocol for a systematic review, meta-analysis, and evidence map
Source: Syst Rev. 2018 Jul 25;7:106. doi: 10.1186/s13643-018-0772-z (PMC6060526; doi:10.1186/s13643-018-0772-z)
Supplement: Supplementary file 1 — Search strategy. Completed search strategy corresponding with this protocol. (PDF 61 kb) [file 13643_2018_772_MOESM1_ESM.pdf]

Search Strategies

**Database**	**Intervention** for MJ
OVID MEDLINE	brief intervention.mp. OR explode motivational interviewing/ OR motivational enhancement.mp. OR motivational interviewing.mp. OR OR SBIRT.mp. OR Psychotherapy, Brief/ OR risk reduction.mp. OR harm reduction.mp. OR Harm Reduction/<br><br>AND<br><br>Marijuana Abuse/ OR Marijuana Smoking/ OR Medical Marijuana/ OR Cannabis/ OR cannabis*.mp. OR marijuana*.mp. OR THC.mp. OR hash.mp. OR marihuana.mp.
EMBASE	brief intervention.mp. OR explode motivational interviewing/ OR motivational interviewing.mp. OR motivational enhancement.mp. OR SBIRT.mp. OR short term psychotherapy/ OR risk reduction.mp. OR OR Risk Reduction/ OR harm reduction.mp. OR OR Harm Reduction/<br><br>AND<br><br>Cannabis/ OR marijuana*.mp. OR Cannabis Addiction/ OR “Cannabis Use”/ OR Cannabis Smoking/ OR Medical Cannabis/ or cannabis*.mp.<br>OR tetrahydrocannabinolic acid/ OR THC.mp. OR tetrahydrocannabinol/ OR hash.mp. OR marihuana.mp.
PsychInfo	Brief intervention.mp. OR explode motivational interviewing/ OR motivational interviewing.mp. OR motivational enhancement.mp. OR SBIRT.mp. OR explode Brief Psychotherapy/ OR risk reduction.mp. OR explode Harm Reduction/ OR harm reduction.mp.<br><br>AND<br><br>Cannabis/ OR marijuana*.mp. OR Marijuana/ OR Marijuana usage/ or cannabis*.mp. OR explode Hashish/ OR THC.mp. OR tetrahydrocannabinol/ OR hash.mp. OR marihuana.mp.<br>.
AMED	Brief intervention.mp. OR motivational interviewing.mp. OR motivational enhancement.mp. OR risk reduction.mp. OR harm reduction.mp.<br><br>AND<br><br>explode Cannabis/ OR cannabis.mp. OR marijuana.mp. OR hash.mp. OR THC.mp. OR marihuana.mp
CINAHL	(MH ‘Psychotherapy, Brief’) OR “brief intervention” OR (MH “Motivational Interviewing”) OR “motivational interviewing” OR “Motivational enhancement” OR “SBIRT” Or “Screening, brief

	intervention, and referral to treatment" OR "Risk Reduction"<br><br>AND<br><br>(MH "Harm Reduction") PR "Harm reduction" or (MH "Cannabis") OR "cannabis" OR (MH "Medical Marijuana") OR "marijuana" OR "marihuana."
Cochrane Clinical Trials (CENTRAL)	Marijuana Motivation (zero results)<br>Cannabis Motivation
Clinical Trials.gov	Cannabis (includes marihuana, cannabinoids, sativex, weed, GW 100, naiximols, Pot)|Child, Adult|studies that accept health volunteers
Current Controlled Trials	Motivational Interviewing OR motivational enhancement or brief intervention<br><br>AND<br><br>Marijuana or cannabis
SAMHSA	Any brief intervention trials
Proquest dissertations	Motivational Interviewing OR motivational enhancement or brief intervention<br><br>AND<br><br>Marijuana or cannabis
WHO portal	Motivational Interviewing OR motivational enhancement or brief intervention<br><br>AND<br><br>Marijuana or cannabis
Google Scholar	Cannabis Brief intervention<br>Cannabis Motivational Interviewing<br>Cannabis Motivational Enhancement<br>Marijuana Brief Intervention<br>Marijuana Motivational Interviewing<br>Marijuana Motivational Enhancement
OpenGrey	Cannabis Brief intervention (1)<br>Cannabis Motivational Interviewing (0)<br>Cannabis Motivational Enhancement (0)<br>Marijuana Brief Intervention (0)<br>Marijuana Motivational Interviewing (0)<br>Marijuana Motivational Enhancement (0)
