# Supplementary material for: Brief interventions for cannabis use in emerging adults: protocol for a systematic review, meta-analysis, and evidence map
Source: Syst Rev. 2018 Jul 25;7:106. doi: 10.1186/s13643-018-0772-z (PMC6060526; doi:10.1186/s13643-018-0772-z)
Supplement: Supplementary file 2 — Screening form. First draft of the screening form (not yet piloted). (PDF 56 kb) [file 13643_2018_772_MOESM2_ESM.pdf]

Screening Form
(Draft 1 – not piloted)

1. POPULATION

Exclude if population restricts to:
- Older adults
- Children
- i.e. exclude if individuals between the ages of 15 and 25 are not included in the study.

2. DESIGN

Is the study design a randomized controlled trial or a quasi-experimental study?
If yes, go to next question.
- If no and indicates another study design (e.g. systematic review, book chapter), exclude.

3. INTERVENTION

i) Is the intervention of interest a brief intervention (i.e. 1 session)?
- If yes or unsure, continue intervention assessment.
- If included additional "booster" sessions but they report outcomes following the single sessions, continue intervention assessment.
- If no, exclude.

ii) Is the intervention focused on illicit substance use or cannabis use?
- If yes or unsure, continue intervention assessment.
- If focused on alcohol or not on substance use, exclude.

4. CONTROL

Is the comparator a within subjects pre-post test, passive-control (wait-list control, no intervention, treatment as usual**), or active control** (i.e. educational sessions, handouts)**?**
- If yes or unsure, continue to outcome assessment.
- If no, exclude.

5. OUTCOME

Is there an outcome on:
- **Cannabis related outcomes**
- **Other substance use related outcomes**
- **Mental health related outcomes**
- **Academic or Occupational outcome**
  - If yes, include.
  - If no, exclude.
