# Supplementary material for: Brief interventions for cannabis use in emerging adults: protocol for a systematic review, meta-analysis, and evidence map
Source: Syst Rev. 2018 Jul 25;7:106. doi: 10.1186/s13643-018-0772-z (PMC6060526; doi:10.1186/s13643-018-0772-z)
Supplement: Supplementary file 3 — Data extraction content. Data that will be extracted from each included full text in this review. (DOCX 92 kb) [file 13643_2018_772_MOESM3_ESM.docx]

**Data Extraction Content**

- **Study ID** (first author name, year, country)
- **Study Design**
- **Demographic Data**
  - Sample size
  - Age (mean, min, max)
  - Context (e.g., university setting, college setting, treatment setting, correctional setting, etc.)
  - Type of screening (e.g. universal vs. targeted)
  - Proportion male
  - Substance use eligibility criteria and baseline use
  - Mental health/illness eligibility criteria and descriptive statistics
  - Mandated or voluntary
- **Risk of bias assessment** (all domains with judgment and quote)
- **Intervention Description**
  - Name of intervention
  - Substance of Focus (i.e. cannabis or general illicit substance use)
  - Number of sessions
  - Length of sessions
  - Delivery method and by whom
  - Fidelity check
  - Content of sessions
    - Screening
    - Personalized feedback (specifically what was provided)
    - Normative feedback
    - Pros/cons
    - Values and Goals Discussion
    - Mental Health discussion
    - Decisional balance and goal setting
    - Additional resources (specifically what was provided)
  - Any adjunctive therapy or booster sessions
- **Comparison Description**
  - Name
  - Length
  - Mode of delivery
  - Description
  - Presence of co-intervention between both intervention and control groups
- **Outcomes**
  - Outcome of interest
  - Measurement used
  - Time of follow-up
  - %Lost to follow-up
  - Was ITT used?
  - Outcome data
    - Continuous [change score and/or post-score mean and standard deviation]
    - Dichotomous [RR or OR and 95% CI]
    - Narrative data
- **Author’s findings** (to compare to our study findings for accuracy)
- **Other Comments**
